# Servant Leadership and Teachers’ Emotional Exhaustion—The Mediation Role of Hindrance Stress and Depersonalization

**DOI:** 10.3390/bs14121129

**Published:** 2024-11-25

**Authors:** Hongchao Wu, Jun Zhao, Shaoping Qiu, Xiuhong Li

**Affiliations:** 1 Center for Educational Evaluation, South China Normal University, Guangzhou 510631, China; wuhc@scnu.edu.cn (H.W.); zhaojun1995@m.scnu.edu.cn (J.Z.); 2 Department of Leadership Studies, Louisiana State University, Shreveport, LA 71115, USA; shaoping.qiu@lsus.edu; 3 School of Art and Design, Guangdong University of Technology, Guangzhou 510006, China

**Keywords:** Chinese teacher, China’s education sector, servant leadership, emotional exhaustion, SEM

## Abstract

**Abstract:** This study aims to explore the impact of school servant leadership on teacher emotional exhaustion in the context of Chinese education, and analyze the mediating effects of teacher hindrance stress and depersonalization. Based on the Job Demand-Control-Support (JDCS) model and servant leadership theory, the research constructs a model for the influence mechanism of school servant leadership on teacher emotional exhaustion and validates it using Structural Equation Modeling (SEM). A total of 3751 primary and secondary school teachers from eight provinces in China participated in this study. The results indicate servant leadership demonstrates a strong negative relationship with emotional exhaustion, with hindrance stress and depersonalization serving as a sequential mediation between servant leadership and emotional exhaustion. This study offers a new perspective on how servant leadership can alleviate emotional exhaustion among teachers, and provides practical insights for optimizing educational management models and enhancing teacher mental well-being.

## 1. Introduction

The teaching profession is generally regarded as one of the most respected, yet it is also one of the most challenging and stressful professions [1]. Emotional exhaustion, one of the key manifestations of job burnout, refers to the prolonged depletion of psychological and emotional resources due to high-intensity work, leading to physical and mental fatigue [2]. Factors such as work burden, administrative pressure, and teacher–student relationships create persistent stress, contributing to the occurrence of emotional exhaustion among teachers [3]. Emotional exhaustion is typically characterized by long-term anxiety, frustration, tension, and fear [4]. Severe emotional exhaustion can lead to a decline in teachers’ professional identity, ultimately affecting teaching effectiveness and student performance [5]. Therefore, effective support for teachers, especially in alleviating their emotional burden and work-related stress, is essential for promoting their mental well-being and professional development.

Servant leadership, a leadership paradigm centered on service and motivation, emphasizes prioritizing employees’ needs and well-being, empowering them through empathy and humility to promote overall team development [6]. This leadership approach advocates using service to others as a motivating tool to create a harmonious and secure work environment, thereby effectively enhancing employee productivity [7,8]. In recent years, researchers have increasingly focused on the development issues of servant leaders themselves. Due to the other-oriented nature of servant leadership, negative feedback from employees may emotionally and cognitively burden leaders, leading to their own emotional exhaustion [9]. Conversely, In the field of education, particularly in higher education, servant leadership helps promote teachers’ innovative achievements, thus enhancing the overall scientific and technological innovation effectiveness of universities [10].

Despite the growing body of research highlighting the positive impact of servant leadership, the specific mechanisms through which it affects teachers’ emotional exhaustion have not been fully explored. Existing studies show that servant leadership, by creating an environment of care, support, and service, can significantly alleviate teachers’ emotional exhaustion, thereby improving their mental health and professional well-being [11]. Psychological safety may mediate this relationship by providing teachers with a sense of security to better cope with emotional demands at work [12]. According to servant leadership theory, external support provided by school leaders (such as recognition, rewards, and career development opportunities) can temporarily boost teachers’ motivation, but sustained motivation mainly comes from teachers’ intrinsic qualities (such as self-efficacy and sense of belonging). Only when internal and external support interact can teachers rely on their intrinsic drive to self-regulate, engage more actively in their work, enhance professional identity, and promote professional growth and development [13]. Therefore, when exploring the relationship between servant leadership and emotional exhaustion, it is crucial to consider both external and internal sources of stress faced by teachers.

The primary reason for teachers’ poor mental health is their prolonged exposure to high-intensity work stressors [1]. However, not all work stress is negative. Cavanaugh classifies stressors in the work environment into challenge stress and hindrance stress. The former refers to positive stress that helps teachers grow, while the latter refers to negative stress that hinders goal achievement and limits teachers’ success. Hindrance stress usually exceeds an employee’s personal control and cannot be alleviated effectively through self-regulation [14]. Teachers’ hindrance stress often arises from external factors such as educational policies, job tasks, resource support, and administrative affairs [15]. A large body of research has shown that hindrance stress negatively impacts teachers’ mental health [16], self-efficacy [17], and job engagement [18], and increases turnover intentions [19].

Teachers’ social relationships with students, colleagues, and school leaders are considered sources of emotional support [20]. Positive social relationships not only enhance teachers’ professional well-being but also effectively improve teaching quality [21]. However, the complex and dynamic social relationships in the workplace often lead to conflicts, which may result in teachers developing depersonalization tendencies [22]. Depersonalization is characterized by teachers adopting a negative attitude toward students and colleagues, emotionally distancing themselves [2]. This tendency negatively impacts teachers’ motivation to teach, leading to conflicts with students and fostering disruptive behaviors [23].

Hindrance stress and depersonalization are key factors influencing teachers’ emotional states and mental health. Effectively managing these two factors has become an essential responsibility for school administrators. Servant leadership can significantly reduce the negative effects of hindrance stress by empowering teachers, building trust, and optimizing workload distribution [24]. However, depersonalization has inherent characteristics, which not only are influenced by hindrance stress [25] but are also closely related to teachers’ intrinsic motivation [26]. Only when teachers have a high level of autonomy can school leaders’ support effectively alleviate their depersonalization tendencies [27]. Therefore, servant leadership should stimulate teachers’ intrinsic drive through internal and external support mechanisms, promoting their self-regulation abilities to alleviate emotional exhaustion.

This study uses hindrance stress and depersonalization as mediating variables between servant leadership and emotional exhaustion in teachers, constructing a sequential mediation model, and verifying the relationships between variables using Structural Equation Modeling (SEM). The contribution of this research lies in its verification of the role of servant leadership in alleviating teachers’ emotional exhaustion in the context of the Chinese educational system. Based on the Job Demands-Control-Support (JDCS) model and servant leadership theory, this study proposes the mechanism through which servant leadership affects teachers’ emotional exhaustion. This study argues that servant leadership, through internal and external support, reduces teachers’ hindrance stress and depersonalization, stimulates intrinsic motivation, and promotes self-regulation of emotional states, thereby effectively alleviating emotional exhaustion. This mechanism provides a new perspective for understanding servant leadership in alleviating teachers’ emotional exhaustion and offers practical insights for optimizing educational management models and enhancing teachers’ mental health.

## 2. Theoretical Background and Research Hypotheses

### 2.1. Theoretical Background

#### 2.1.1. Job Demands-Control-Support (JDCS) Model

The Job Demands-Control-Support (JDCS) model integrates three fundamental psychological dimensions: job demands, job control, and social support. Job demands refer to factors such as workload, time pressure, and role conflict, which contribute to the pressures experienced by employees. Job control encompasses the degree of autonomy employees have over their tasks and the ability to use their skills effectively in the workplace [28]. Social support pertains to the resources provided by leaders, colleagues, and personal networks that help employees cope with job-related stress [29]. The JDCS model posits that when job demands are high, job control is limited, and social support is insufficient, employees face heightened psychological stress [30]. Some studies have indicated the link between a lack of social support and increased job burnout [31,32,33].

#### 2.1.2. Servant Leadership Theory

Servant leadership, introduced by Greenleaf [34], is founded on the principle that leadership arises from the desire to serve others. Leaders in this model prioritize meeting the needs of their followers, fostering a sense of trust and collaboration. Servant leadership theory suggests that, while external incentives like compensation may provide temporary motivation, intrinsic motivators—such as self-efficacy and a sense of belonging—play a more enduring role in sustaining employee engagement and performance [13]. Therefore, the servant leadership model underscores that leaders can cultivate intrinsic motivation by initially providing external support mechanisms, such as recognition and professional development opportunities. This leadership style has been shown to promote both personal and professional development, making it particularly relevant for addressing the psychological well-being of teachers, who often face significant emotional challenges in their work [35,36,37].

The JDCS model highlights the critical role of social support in helping employees cope with work-related stress. As a novel form of social support, servant leadership, by providing necessary resources and services, can effectively alleviate employee stress. Research indicates a significant correlation between teachers’ stress and emotional exhaustion [38,39]. Therefore, the JDCS theory provides theoretical support for understanding the impact of servant leadership on teachers’ emotional exhaustion.

Simultaneously, servant leadership theory emphasizes the importance of both external and internal support in stimulating teachers’ intrinsic motivation, thereby enhancing their self-regulation capacity. Building on this theoretical framework, the current study explains how servant leadership impacts teachers’ emotional exhaustion by reducing their hindrance stress and depersonalization tendencies, while increasing external support and internal incentives.

The JDCS model and servant leadership theory together provide a strong theoretical foundation for exploring the relationship between servant leadership and teachers’ emotional exhaustion, as well as the underlying mechanisms at play. This dual framework allows for a comprehensive understanding of how servant leadership can mitigate emotional exhaustion in teachers by fostering a supportive environment that promotes intrinsic motivation and emotional self-regulation.

### 2.2. Servant Leadership and Teachers’ Emotional Exhaustion

After entering the education sector, teachers gradually form and deepen their professional identity through specific experiences in classrooms and schools. However, emotional exhaustion hinders the internalization of professional identity, gradually weakening teachers’ sense of belonging to the education profession [40]. The lack of professional identity leads teachers to attribute emotional exhaustion to inherent and unchangeable personal traits. This tendency may prompt teachers to seek alternative career paths when faced with professional challenges, thereby exacerbating teacher turnover rates [41].

According to the JDCS model, social support plays a critical role in mitigating emotional exhaustion, and leaders are often a key source of such support in educational settings. Servant leadership, which emphasizes putting employees’ needs above the leader’s self-interest and fostering shared power and care, contrasts with more traditional leader-centric models, promoting teacher well-being [42]. Empirical research has shown that servant leadership is effective in reducing emotional exhaustion [43], and school principals who adopt this leadership style positively influence teachers’ emotional commitment and mental health [44].

Thus, the following hypothesis is proposed:

**H1.**  *Perceived servant leadership is negatively related to teachers’ emotional exhaustion.*

### 2.3. The Mediating Role of Teachers’ Hindrance Stress in the Relationship Between Servant Leadership and Emotional Exhaustion

Hindrance stress refers to stressors that impede employees’ career development and goal achievement, such as role conflicts, organizational bureaucracy, and job insecurity [2]. In the school environment, excessive administrative tasks, frequent evaluations, and rigid policies exacerbate teachers’ long-term hindrance stress. Such stress increases depressive symptoms among teachers, further impacting their physical and mental health [45]. Moreover, high-intensity work stress often leads to psychological tension and fatigue, resulting in decreased attention, insomnia, depression, and even cardiovascular issues [46]. Servant leadership can effectively alleviate hindrance stress by streamlining work processes and implementing human-centered policies, thereby enhancing teachers’ organizational identification [47].

Hindrance stress drains teachers’ emotional resources, leaving them vulnerable to exhaustion due to the persistent demands of their roles. According to the JDCS model, teachers exposed to high job demands in conjunction with low job control, are at a significantly elevated risk of emotional exhaustion. Specifically, high-intensity hindrance stressors such as heavy administrative tasks, challenges in managing student behavior, or unrealistic job expectations constrain teachers’ autonomy and decision-making power, continuously depleting their emotional and cognitive resources. This, in turn, exacerbates the negative impact of work-related stress on their physical and mental well-being [32]. Research supports this relationship, showing that hindrance stress is positively correlated with emotional exhaustion [48].

Therefore, the following hypotheses are proposed:

**H2.**  *Perceived servant leadership is negatively related to teachers’ hindrance stress.*

**H3.**  *Teachers’ hindrance stress is positively related to emotional exhaustion.*

**H4.**  *Hindrance stress mediates the relationship between perceived servant leadership and teachers’ emotional exhaustion.*

### 2.4. The Mediating Role of Teachers’ Depersonalization in the Relationship Between Servant Leadership and Emotional Exhaustion

Depersonalization, a key component of teacher burnout, refers to the emotional distancing teachers develop toward their students in response to the psychological demands of their work [49]. This sense of alienation may lead teachers to develop negative attitudes toward students and a lack of empathy, further distancing teacher–student relationships and potentially triggering disruptive classroom behavior among students [27]. Effective leadership, particularly servant leadership, can reduce teachers’ tendencies toward depersonalization [50,51]. School leaders can foster a positive work environment, bridging the gap between teachers and leaders, colleagues and students, while inspiring teachers’ intrinsic motivation to engage more actively in their work. Furthermore, emotional exhaustion is closely linked to depersonalization. When teachers invest significant emotional energy without sufficient rewards or recognition, the imbalance between effort and reward depletes emotional resources, gradually alienating teachers from others and exacerbating tendencies toward depersonalization [52].

Thus, the following hypotheses are proposed:

**H5.**  *Perceived servant leadership is negatively related to teachers’ depersonalization.*

**H6.**  *Depersonalization is positively related to emotional exhaustion.*

**H7.**  *Depersonalization mediates the relationship between perceived servant leadership and teachers’ emotional exhaustion.*

### 2.5. The Sequential Mediating Role of Hindrance Stress and Depersonalization in the Relationship Between Servant Leadership and Emotional Exhaustion

Under the dual pressures of family and workplace demands, teachers often face role conflicts, with the accumulation of hindrance stress potentially intensifying tendencies toward depersonalization, which in turn leads to emotional exhaustion [53]. According to servant leadership theory, providing internal and external support services can stimulate teachers’ intrinsic motivation, enabling them to achieve self-regulation. Therefore, school leaders can effectively promote teachers’ emotional self-regulation by reducing hindrance stress and depersonalization.

Therefore, the following hypotheses are proposed:

**H8.**  *Hindrance stress is positively related to depersonalization.*

**H9.**  *Hindrance stress and depersonalization serve as sequential mediators in the relationship between servant leadership and teachers’ emotional exhaustion.*

**Figure 1:**
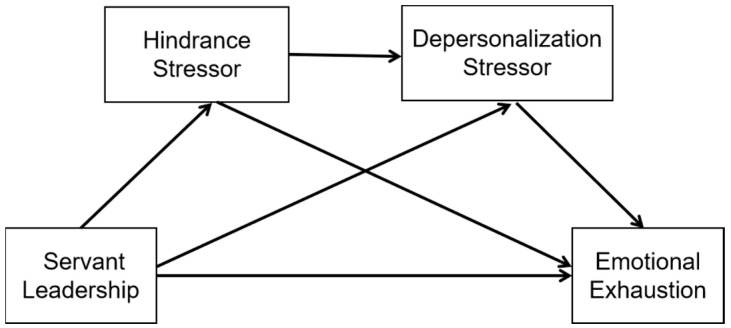
Conceptual model.

Based on the discussion of the above research hypotheses, this study constructs a conceptual model of the mechanism by which servant leadership influences teachers’ emotional exhaustion, as shown in Figure 1.

## 3. Research Design

### 3.1. Participants and Procedures

This study was conducted in collaboration with municipal education bureaus across eight provinces in China: Guangdong, Guangxi, Jiangxi, Jilin, Zhejiang, Henan, Hunan, and Xinjiang. The data were collected from elementary, middle, and high school teachers between February and April 2024. The research team provided detailed explanations to these bureaus regarding the study’s background, objectives, and significance. After obtaining consent from both the participating schools and individual teachers, the education bureaus coordinated the distribution of the questionnaires. Data collection was facilitated through “Wenjuanxing”, a widely used online survey platform in China. Education bureaus distributed WeChat messages containing links to the survey, allowing teachers to participate voluntarily. Survey items from various constructs were randomly distributed during data collection. The confidentiality and anonymity of respondents were ensured.

To ensure the semantic accuracy and cultural appropriateness of the survey instrument, English-major teachers conducted a back-translation of all scales. Additionally, three university professors with expertise in relevant fields were consulted to review and assess the questionnaire. Based on their feedback, we revised the wording of certain questionnaire items to better align with the context of the Chinese education system, thereby enhancing the clarity and cultural relevance of the questionnaire. A pilot test was conducted with 60 teachers in Guangzhou prior to the formal survey launch. The feedback from this pilot informed further adjustments to the scales, ensuring that participants could clearly understand and engage with the survey content.

A total of 5260 elementary, middle, and high school teachers were invited to complete the online questionnaire. After rigorous data screening, which involved the removal of duplicate, incomplete, and anomalous responses, a total of 3751 valid questionnaires were retained, yielding a final effective response rate of 71.31%.

The demographic characteristics of the participating teachers are shown in Table 1. Among them, 75.34% were female teachers, and 24.66% were male teachers. The average age of participants was 37.34 years (*SD* = 9.09). The average teaching experience among respondents was 14.7 years (*SD* = 10.06). Geographically, 65.05% of the teachers were from urban areas, 18.63% from towns, and 16.32% from rural locations. By educational stage, 51.83% were elementary school teachers, 21.70% were middle school teachers, and 26.47% were high school teachers. Furthermore, 87.79% of the respondents were employed in public schools.

### 3.2. Measures

#### 3.2.1. Perceived Servant Leadership

The Perceived Servant Leadership Scale was adapted from the short version of the Servant Leadership Behavior Scale (SLBS-6) developed by Sendjaya et al. [54]. The original scale consists of six items, with a sample item being “My principal uses their power to serve others, not for personal gain”. After testing the measurement model, four items with standardized factor loadings above 0.5 were retained. The Cronbach’s alpha coefficient for this scale was 0.85. A five-point Likert scale was employed, ranging from 1 (strongly disagree) to 5 (strongly agree), with higher scores indicating a stronger perception of servant leadership characteristics among school leaders.

#### 3.2.2. Hindrance Stress

The Hindrance Stress Scale was adapted from the stress scale developed by LePine et al. [55]. The original scale contains ten items, with a sample item being “I often receive conflicting instructions and expectations from different leaders”. After testing the measurement model, six items were retained. The Cronbach’s alpha coefficient for this scale was 0.86. A five-point Likert scale was utilized, where higher scores reflect greater levels of hindrance stress perceived by teachers.

#### 3.2.3. Depersonalization

The Depersonalization Scale was derived from the depersonalization subscale of the Maslach Burnout Inventory MBI-ES [56]. The original scale consists of five items, with a sample item being “Since starting this job, I have become more emotionally distant from people”. Following the measurement model test, three items were retained. The Cronbach’s alpha coefficient for this scale was 0.78. A five-point Likert scale was applied, with higher scores indicating a greater degree of emotional detachment from students.

#### 3.2.4. Teacher Emotional Exhaustion

The Teacher Emotional Exhaustion Scale was adapted from the emotional exhaustion subscale of the Maslach Burnout Inventory MBI-ES [56]. The original scale comprises nine items, with a sample item being “I feel frustrated by my work”. After testing the measurement model, four items were retained. The Cronbach’s alpha coefficient for this scale was 0.84. A five-point Likert scale was used, with higher scores indicating greater emotional exhaustion experienced by teachers due to their work.

### 3.3. Data Analysis

Data analysis was conducted using SPSS version 27.0 to screen for outliers and missing data, followed by a test for common method bias. Descriptive statistics and correlation analysis were performed to assess distribution trends and relationships among variables. Subsequently, confirmatory factor analysis (CFA) was conducted using Mplus version 8.0 to evaluate the reliability, construct validity, and discriminant validity of the scales. Finally, Structural Equation Modeling (SEM) was employed to test the research hypotheses, and the Bootstrap method was utilized to explore the sequential mediating effects of teacher depersonalization and hindrance stress. We used SEM instead of multiple regression because SEM accounts for the measurement errors. The estimates of the effect size produced have no statistical bias, thus generating more accurate results and boosting statistical power [57]. PLS was not utilized in this study because the SmartPLS 4.0 software was unavailable to us.

## 4. Results

### 4.1. Common Method Bias

When using self-reported questionnaires to collect data, common method variance (CMV) is a potential concern [58]. This study constructed a four-factor model (Model 1) consisting of servant leadership, hindrance stress, depersonalization, and emotional exhaustion. We then compared the four-factor model with alternative models that combined various constructs to evaluate the potential impact of CMV. As shown in Table 2, the four-factor model (Model 1) demonstrated better fit indices compared to the other models, with all indices meeting acceptable standards.

In addition, the Harman single-factor test showed that the variance explained by the first factor was 42.85%, which is below the 50% threshold. These findings indicate that common method bias is unlikely to have a significant impact on the results of this study [59].

### 4.2. Descriptive Statistics

This study conducted descriptive statistical analysis on the variables of perceived service-oriented leadership, depersonalization, hindering stress, and emotional exhaustion. According to the results presented in Table 3, the absolute values of skewness for all variables were less than 1, and the absolute values of kurtosis were less than 1, indicating that the sample data approximate a normal distribution. The mean score for emotional exhaustion was 3.03 (*SD* = 0.93), which was a moderate level. The mean score for hindering stress was 3.21 (*SD* = 0.84), which was above the moderate level. The mean score for depersonalization was 2.30 (*SD* = 0.90), which was below the moderate level.

The mean score for perceived servant leadership was 3.23 (*SD* = 0.87), which was above the moderate level. These results indicate that the surveyed elementary, middle, and high school teachers experience considerable hindering stress and show signs of emotional exhaustion. However, the level of teacher depersonalization is relatively low, suggesting that they still maintain a high degree of care for their students. Additionally, most teachers perceive that school leadership is providing necessary resources and services, reflecting the qualities of service-oriented leadership.

### 4.3. Reliability and Validity Testing and Correlation Analysis

This study evaluated the reliability of the scales through Composite Reliability (CR) coefficients and assessed their validity using convergent validity and discriminant validity. The results in Table 4 indicate that the CR values of the latent variables range from 0.79 to 0.85, all exceeding 0.7, demonstrating a high level of internal consistency. The standardized factor loadings for each variable are all greater than 0.6, and the Average Variance Extracted (AVE) for each variable is above 0.5, indicating that the scales possess good convergent validity. Furthermore, the square root of the AVE for each variable is greater than its correlations with other variables, confirming the scales have satisfactory discriminant validity.

In addition, the results of the correlation analysis reveal significant negative correlations between perceived servant leadership and emotional exhaustion (*r* = −0.38, *p* < 0.01), depersonalization (*r* = −0.36, *p* < 0.01), and hindrance stress (*r* = −0.51, *p* < 0.01). Emotional exhaustion is significantly positively correlated with depersonalization (*r* = 0.66, *p* < 0.01), while hindering pressure shows significant positive correlations with emotional exhaustion (*r* = 0.69, *p* < 0.01) and depersonalization (*r* = 0.55, *p* < 0.01).

### 4.4. Hypothesis Testing

This study utilized Structural Equation Modeling (SEM) to examine the relationships among the variables of servant leadership, hindering pressure, depersonalization, and emotional exhaustion. The results indicate that the model demonstrates good fit (*χ*^2^ = 1156.85, *df* = 113.00, *RMSEA* = 0.05, *GFI* = 0.97, *TLI* = 0.96, *SRMR* = 0.03). As shown in Table 5, servant leadership has a significant negative impact on teachers’ hindering pressure (*β* = −0.56, *p* < 0.001), depersonalization (*β* = −0.05, *p* < 0.01), and emotional exhaustion (*β* = −0.06, *p* < 0.01). Furthermore, hindering pressure significantly positively influences depersonalization (*β* = 0.50, *p* < 0.001) and emotional exhaustion (*β* = 0.62, *p* < 0.001), while depersonalization also significantly positively affects emotional exhaustion *(β* = 0.86, *p* < 0.001). Therefore, Hypotheses 1 to 6 have been validated. Additionally, the coefficient of determination (R^2^) is used to assess the explanatory power of the model for latent variables. The R^2^ value for teacher emotional exhaustion is 0.80, which exceeds 0.67, indicating that the model has a high explanatory power for emotional exhaustion. The R^2^ value for teacher depersonalization is 0.43, and for hindrance stress, the R^2^ value is 0.34, both of which exceed 0.33, suggesting that the model has a moderate explanatory power for these two variables.

### 4.5. Mediation Effect Testing

This study employed Bootstrap testing methods to examine the mediating role of hindering pressure between depersonalization and emotional exhaustion. A total of 1000 sampling iterations were set, calculating the point estimates of mediation effects and the 95% confidence intervals for each sampling. The testing results are detailed in Table 6.

In the examination of how servant leadership impacts teachers’ emotional exhaustion, hindrance stress is identified as a significant mediating factor, with a confidence interval (CI) of 95% ranging from −0.40 to −0.31, thus supporting the validation of Hypothesis H7. The estimated point value for the mediating effect of hindrance stress is −0.35, which represents 50.72% of the total effect (mediating effect estimate/total effect estimate). Additionally, the direct effect of servant leadership on emotional exhaustion is significant (95% CI: −0.10 to −0.02), suggesting that hindrance stress plays a partial mediating role in this relationship.

Moreover, depersonalization emerges as another mediating factor in the influence of servant leadership on teachers’ emotional exhaustion (95% CI: −0.07 to −0.01), corroborating the validation of Hypothesis H8. The estimated point value for the mediating effect of depersonalization is −0.04, accounting for 5.80% of the total effect, thereby indicating partial mediation.

Both hindrance stress and depersonalization collectively exhibit a sequential mediation effect in the relationship between servant leadership and teachers’ emotional exhaustion, with a 95% CI ranging from −0.27 to −0.21, which further substantiates the validity of Hypothesis H9. The combined estimated point value for the mediating effects of hindrance stress and depersonalization is −0.24, which constitutes 34.78% of the total effect.

## 5. Discussion

This study explores the interrelationships between servant leadership, hindrance stress, depersonalization, and emotional exhaustion within China’s basic education system. The findings indicate that servant leadership can effectively alleviate teacher emotional exhaustion, with hindrance stress and depersonalization playing a mediating role in the influence of servant leadership on teacher emotional exhaustion.

Firstly, this study reveals that school servant leadership has a significant negative relationship with teacher emotional exhaustion, suggesting that when school leaders exhibit servant leadership behaviors, they can effectively alleviate teacher emotional exhaustion. This conclusion is consistent with related studies in other industries [9,35]. However, research on the relationship between servant leadership and emotional exhaustion in the Chinese education system is relatively scarce, and this study expands the research scope in this field, providing new empirical support. Moreover, previous studies have often linked servant leadership to employee burnout, with emotional exhaustion being a core factor in burnout [49]. This study further refines existing research by focusing on teacher emotional exhaustion and exploring the supportive role of school leadership in managing teachers’ emotional states.

In recent years, excessive emphasis on academic performance and examination rates in China’s basic education system has exacerbated the pressure on teachers. Teachers also face complex social relationships with leaders, colleagues, parents, and students. Balancing these multiple relationships consumes large amounts of emotional resources, leading teachers to feel increasingly fatigued and emotionally exhausted. Additionally, the gap between society’s respect for the teaching profession and the actual working environment causes many teachers to experience psychological disillusionment after entering the profession, leading to a decline in professional identity and an increase in negative emotions. This study shows that school leaders’ servant leadership behaviors can effectively alleviate teacher emotional exhaustion. Servant leaders typically invest more effort in supporting teachers and grant them more decision-making power [60]. When teachers feel fairly treated and enjoy autonomy in their work, their job satisfaction significantly improves [36], and their willingness to actively engage in school development increases, thereby reducing emotional resource depletion. This perspective offers important practical guidance for schools in building teacher teams and managing professional identity development.

Secondly, this study finds that school servant leadership has a significant negative impact on teachers’ hindrance stress and depersonalization tendencies, consistent with existing research [47]. According to JDCS theory, high levels of social support can effectively alleviate employees’ work stress. This study reveals that servant leadership, by providing resource support, reduces the hindrance stress caused by external factors in teachers’ career development, offering empirical support for JDCS theory. Additionally, this study shows that school servant leadership can effectively reduce teachers’ depersonalization tendencies, which helps motivate teaching and foster positive social relationships. This result aligns with studies in other industries and extends its application to Chinese basic education teachers [61]. Furthermore, this study finds a significant positive correlation between teachers’ hindrance stress and depersonalization tendencies. This finding is rarely addressed in research and provides a new perspective on the relationship between work stress and emotional exhaustion among teachers, further enriching the research in this area.

In addition to their routine teaching responsibilities, teachers are often required to dedicate substantial time and energy to administrative tasks, such as holding leadership positions within the school, serving as homeroom teachers, and managing duties like teaching evaluations, event planning, and extracurricular activities. These responsibilities typically involve coordinating school operations, managing classes, communicating with parents, and organizing after-school events. When teachers lack autonomy or decision-making authority in these matters, their hindrance stress can increase significantly [30]. Research indicates that hindrance stress accelerates the depletion of emotional resources, making teachers more susceptible to emotional exhaustion. Thus, excessive administrative workloads and complex management duties not only contribute to higher hindrance stress but also lead to emotional exhaustion under prolonged heavy workloads. Servant leadership offers an effective approach to alleviating the hindrance stress associated with administrative demands. By empowering teachers, encouraging their involvement in democratic decision-making, and ensuring an equitable distribution of tasks, servant leadership can mitigate the negative impacts of administrative burdens [44]. This leadership style not only helps reduce teachers’ emotional exhaustion but also introduces innovative solutions for school management. It fosters a supportive and collaborative work environment, enhancing teachers’ job satisfaction and professional well-being.

Student behavior issues, such as lack of focus, poor classroom discipline, or mental health challenges, coupled with parents’ high expectations—such as excessive demands for academic performance or teaching quality—subject teachers to intense pressure in their interactions with students and parents. At the same time, heavy family responsibilities, such as raising children and caring for elderly parents, further strain teachers’ time and energy. These overlapping role conflicts significantly exacerbate teachers’ tendencies toward depersonalization, causing them to become emotionally detached from others and exhibit indifferent attitudes in their work [49]. Research further indicates that servant leadership can effectively alleviate teachers’ tendencies toward depersonalization. It is recommended that school leaders adopt servant leadership behaviors more extensively in the future. By offering personalized care and targeted motivational strategies, leaders can deeply understand and respect teachers’ emotions, perspectives, and values, thereby fostering a positive and uplifting work environment. Additionally, emphasis should be placed on facilitating effective communication and collaboration between teachers, school administrators, and colleagues. Such initiatives can help teachers regain their professional enthusiasm and sense of accomplishment. Not only does this reduce depersonalization tendencies among teachers, but it also establishes a solid foundation for their long-term professional well-being and career development.

Thirdly, hindrance stress and depersonalization mediate the impact of school servant leadership on teacher emotional exhaustion. This study shows that, although school servant leadership has a significant statistical impact on teacher emotional exhaustion, the direct effect is weak (Point Estimate: −0.06), indicating that school servant leadership may influence teacher emotional exhaustion through other mediating variables. All three proposed mediating pathways were verified. Among them, hindrance stress has the largest effect size in the indirect effect of servant leadership on teacher emotional exhaustion (Point Estimate: −0.35), indicating that school servant leadership can effectively alleviate emotional exhaustion by reducing teachers’ hindrance stress, which aligns with previous research [47]. Additionally, this study finds that teachers’ depersonalization tendencies also play an indirect role in the effect of servant leadership on emotional exhaustion, but the effect size is weak (Point Estimate: −0.04). This phenomenon may be due to the fact that servant leadership only provides external support and has limited impact on motivating teachers’ intrinsic motivations and depersonalization tendencies. Previous studies have also indicated a relationship between teachers’ intrinsic motivation and depersonalization [26,27].

The theory of servant leadership emphasizes that managers with servant leadership traits should provide internal and external support to inspire teachers’ intrinsic motivation and help them realize their self-worth. The research findings show that obstructive stress and depersonalization tendencies act as chain mediators in the impact of servant leadership on teacher emotional exhaustion (Point Estimate: −0.24). Notably, these three variables collectively explain 80% of the variation in teacher emotional exhaustion (R^2^: 0.80). This result suggests that school servant leadership reduces external obstructive stress, weakens teachers’ depersonalization tendencies, and stimulates their intrinsic motivation, thereby encouraging teachers to regulate their emotional state. This study makes a novel contribution by providing empirical support for servant leadership theory and constructing an outward-to-inward pathway in teacher psychological adjustment, further enriching the understanding of how servant leadership impacts employees’ emotional exhaustion.

Currently, China’s primary and secondary education system has made significant progress in areas such as teaching environment, training and professional development opportunities, and compensation, with teachers receiving the necessary resources and support, and their professional competencies improving. However, this study’s findings show that teachers in China’s primary education system still face moderate levels of emotional exhaustion. This phenomenon can be explained by the current study: specifically, schools’ servant leadership tends to focus too much on external support for teachers while neglecting to stimulate their intrinsic motivation. The lack of sufficient care for teachers’ well-being leads to limited development opportunities and a sense of achievement amidst heavy workloads, which exacerbates emotional exhaustion. This finding has important implications for teacher workforce development and career health. In the future, while providing social support to teachers, governments and schools should cultivate a culture of respect for teachers, increase recognition and promotion efforts, and enhance teachers’ sense of mission, achievement, and well-being. By stimulating their intrinsic motivation, they can unlock personal potential and work enthusiasm, promoting the sustainable development of the teaching profession.

Fourth, teaching motivation and teacher–student relationships may be potential mechanisms through which servant leadership affects teacher emotional exhaustion. This study found that servant leadership can reduce depersonalization tendencies by stimulating teachers’ intrinsic motivation, but it has not yet revealed the specific factors that influence teachers’ intrinsic motivation. Teaching motivation is a key predictor of teachers’ mental health [62]. Therefore, future research should focus on how school servant leadership stimulates teachers’ teaching motivation through internal support. Teachers with strong intrinsic motivation are more likely to devote more time and energy, realizing their self-worth in professional development. Additionally, teachers’ depersonalization tendencies are closely related to teacher–student relationships [27]. Positive teacher–student relationships encourage teachers to actively attend to and meet students’ needs, thereby increasing student engagement [63], ultimately improving teaching efficiency.

## 6. Implications

This study offers several theoretical contributions. First, it significantly adds to the existing literature on servant leadership and teacher emotional exhaustion. As previously discussed, research examining the overall relationship between servant leadership, teachers’ hindrance stress, depersonalization, and emotional exhaustion remains relatively scarce. By exploring the interactions among these variables, this study deepens our understanding of the dynamic relationship between servant leadership and emotional exhaustion within the teaching profession.

Second, this study elucidates how servant leadership influences teacher emotional exhaustion within the context of the Chinese education sector, testing the partial mediating roles of teacher hindrance stress and depersonalization in this process. Through the construction of a theoretical model that explains the relationship between servant leadership and teacher emotional exhaustion, the research reveals the mechanisms by which servant leadership alleviates teacher emotional exhaustion by reducing hindrance stress and depersonalization. This study provides a novel perspective on the impact of servant leadership on teacher emotional exhaustion, enriching the theoretical framework surrounding this area of research.

This study also has practical implications. First, the research supports the effectiveness of servant leadership within the Chinese teaching profession. The results indicate that school leaders who exhibit greater servant leadership behaviors can significantly reduce teachers’ hindrance stress, diminish tendencies toward depersonalization, and alleviate emotional exhaustion. In the past, school leaders often relied on external incentives to improve teacher performance, but these approaches had limited success in fostering intrinsic motivation. This study further finds that reducing hindrance stress stimulates teachers’ intrinsic motivation, which not only lessens depersonalization but also effectively mitigates emotional exhaustion. Therefore, future servant school leaders should place greater emphasis on cultivating teachers’ intrinsic motivation by creating a positive work environment characterized by active student engagement, colleague collaboration and support, and institutional support. Such a positive atmosphere not only increases teacher engagement but also promotes mutual growth between teachers and students, ultimately contributing to the overall improvement in educational quality.

Second, this study demonstrates that teachers’ hindrance stress and depersonalization tendencies serve as mediators in the relationship between servant leadership and teacher emotional exhaustion, further highlighting the negative impact of these factors on teachers’ job performance. Therefore, school leaders should consider moving away from performance-driven evaluation systems that rely solely on singular metrics. Instead, they should adopt a more diversified evaluation approach to alleviate the external hindrance stress faced by teachers. Additionally, schools should encourage teachers to reconnect with the core mission of education, promoting the spirit of educational dedication. This can be achieved by actively recognizing and celebrating teachers who uphold the fundamental values of education, thereby enhancing their sense of professional purpose and pride. By fostering a greater sense of belonging within the teaching community and reducing depersonalization tendencies, teachers can better manage emotional exhaustion, which in turn supports their professional growth and psychological well-being.

## 7. Limitations and Future Studies

This study has several limitations. First, it did not employ a hierarchical linear model (HLM) to investigate the impact of servant leadership at the organizational level on teacher emotional exhaustion. Due to the unavailability of unit-specific information from participating schools, such data could not be analyzed. Future research could benefit from using HLM if these details are accessible, as it would reduce the likelihood of Type I errors and provide more accurate estimations. Second, the cross-sectional design of this study limits its ability to identify causal relationships between the variables. To explore causal connections more robustly, future research should consider adopting a longitudinal design. By conducting repeated measurements over an extended period, longitudinal studies would offer deeper insights into the causal dynamics between variables, thereby contributing more valuable findings to the field. Third, although measures have been taken to control CMV, it may still exist in the data, biasing the estimation of the effects. Future research should rigorously gather data on predictor and outcome variables from different sources, and thus enhance the objectivity and reliability of the conclusions. Last, we focused on emotional exhaustion, a key factor of burnout. However, we are aware that aside from emotional exhaustion, there are other dimensions that constitute burnout, such as reduced professional efficacy and cynicism [2]. Future research can explore these additional dimensions as outcome variables to investigate similar relationships.

## 8. Conclusions

Teachers may face the crisis of job burnout during their professional development, making it crucial to explore ways to minimize the impact of such crises on their career growth and well-being. This study approaches the issue from the perspective of servant leadership and teacher emotional exhaustion. The results indicate that school leaders who exhibit servant leadership behaviors help alleviate emotional exhaustion in teachers during their professional development, with hindrance stress and depersonalization serving as mediators in this relationship. This study highlights that, by providing both internal and external support, servant leadership in schools can effectively reduce teachers’ hindrance stress and depersonalization tendencies, thereby mitigating emotional exhaustion. Therefore, future school administrators should focus on reducing hindrance stress and depersonalization in teachers and adopt more servant leadership behaviors to effectively support teachers’ professional development and mental well-being.

## Figures and Tables

**Table 1:** Demographic variables overview.

Variable	Category	Frequency	Percentage (%)	Variable	Category	Frequency	Percentage (%)
Gender	Male	925	24.66	Teaching Experience	1–10 years	1573	41.94
	Female	2826	75.34		11–20 years	1007	26.85
Age	20–29 years	900	23.99		21–30 years	924	24.63
	30–39 years	1271	33.88		30+ years	247	6.58
	40–49 years	1118	29.81	Education Level	Elementary School	1944	51.83
	50+ years	462	12.32		Middle School	814	21.70
School Location	City/County	2440	65.05		High School	993	26.47
	Town	699	18.63	School Type	Public School	3293	87.79
	Rural	612	16.32		Private School	458	12.21

**Table 2:** Comparison of common method bias CFA results.

MODEL	χ^2^	*df*	RMSEA	GFI	TLI	SRMR
Model 1: SL; HS; DE; EE	1156.85	113.00	0.05	0.97	0.96	0.03
Model 2: SL + HS; DE; EE	4706.07	116.00	0.10	0.85	0.83	0.07
Model 3: SL + HS+DE; EE	6856.83	118.00	0.12	0.78	0.75	0.08
Model 4: SL + HS+DE+EE	7720.49	119.00	0.13	0.75	0.72	0.09

**Table 3:** Descriptive statistics of variables.

Variable	Mean	S.D.	Skewness	Kurtosis
Emotional Exhaustion	3.03	0.93	0.20	−0.54
Depersonalization	2.30	0.90	0.78	0.22
Hinderance Stress	3.21	0.84	−0.04	−0.37
Servant Leadership	3.23	0.87	−0.43	−0.11

**Table 4:** Reliability, validity, and correlation analysis results.

Variable	Std.Estimate	CR	AVE	1	2	3	4
Emotional Exhaustion (1)	0.72~0.79	0.84	0.56	0.75			
Depersonalization (2)	0.61~0.82	0.79	0.56	0.66 **	0.75		
Hindrance Stress (3)	0.62~0.79	0.86	0.51	0.69 **	0.55 **	0.71	
Servant Leadership (4)	0.71~0.83	0.85	0.59	−0.38 **	−0.36 **	−0.51 **	0.77

Note: The values in the shaded area represent the square root of each variable’s AVE, while the values below the shaded area indicate the correlation coefficients among the variables. ** *p* < 0.01 (two-tailed).

**Table 5:** Results of path coefficient testing for research hypotheses.

Independent Variable	Dependent Variable	Unstd. Estimate	S.E.	R^2^
Servant Leadership	Hindrance Stress	−0.56 ***	0.02	0.34
Servant Leadership	Depersonalization	−0.05 **	0.20	0.43
Hindrance Stress		0.50 ***	0.02	
Servant Leadership	Emotional Exhaustion	−0.06 **	0.02	0.80
Hindrance Stress		0.62 ***	0.03	
Depersonalization		0.86 ***	0.04	

Note: *** *p* < 0.001, ** *p* < 0.01 (two-tailed).

**Table 6:** Mediation effect testing.

Path	Point Estimate	S.E.	Bias Corrected 95% CI	
			Lower	Upper
Total Effect	−0.69	0.03	−0.75	−0.63
Total indirect Effect	−0.63	0.03	−0.69	−0.57
Direct Effect	−0.06	0.02	−0.10	−0.02
SL->HS->EE	−0.35	0.02	−0.40	−0.31
SL->DE->EE	−0.04	0.02	−0.07	−0.01
SL->HS->DE->EE	−0.24	0.02	−0.27	−0.21

Note: HS = Hindrance Stress; SL = Servant Leadership; DE = Depersonalization; EE = Emotional Exhaustion.

## Data Availability

Data are available by contacting the corresponding author.
